# Usage of a “Unique” Digital Surgical Guide for Simultaneous Crown Lengthening, Implant Placement, and Soft Tissue Thickening in the Esthetic Zone

**DOI:** 10.1155/crid/7166599

**Published:** 2025-08-18

**Authors:** Bachar Husseini, Nabil Ghosn, Ranim Abou Chakra, Patrick El Sayegh, Marie Jose Merhej, Hussein Ftouni, Carlos Moussallem, Anton Friedmann

**Affiliations:** ^1^Dental Square Clinic, Beirut, Lebanon; ^2^Dental Studio, Byblos, Lebanon; ^3^Improdentalab, Beirut, Lebanon; ^4^Department of Periodontology, Faculty of Health, Witten/Herdecke University, Witten, Germany

**Keywords:** altered passive eruption, CBCT, crown lengthening, digital dentistry, surgical guide

## Abstract

Managing complex esthetic cases involving crown lengthening and anterior implant placement in compromised ridges often requires a staged, multidisciplinary approach. Such treatment modality is often lengthy and demanding for both the patient and the practitioner. With the rise of digital planning software, treatment can now be streamlined by providing a preview of the expected outcome. The “unique” surgical guide is the first of its kind to enable practitioners to simultaneously perform patient-tailored crown lengthening and implant placement. This novel concept may enhance the patient's treatment experience by reducing surgical interventions and simplifying the prosthodontic workflow.

## 1. Introduction

Achieving satisfactory esthetic outcomes in cases with a high smile line and altered passive eruption, along with anterior hard and soft tissue deficiencies, is usually challenging, particularly if both teeth and implant-supported restorations are involved [1, 2]. Such procedures require multidisciplinary and staged planning and execution, leaving no room for error [1, 2]. Moreover, a thorough discussion with the patient about the potential outcomes is mandatory to set realistic goals [3].

Normally, to reduce the complexity of a case, the conventional approach divides the treatment sequence into four phases. Initially, it begins with regeneration of the lost tissue before passing to crown lengthening. Accordingly, the obtained gingival level will later demarcate the exact prosthetically driven position of the desired dental implant [4] followed by implant placement and final restoration of the entire case [5]. It is considered a time-consuming procedure that involves numerous visits and long chairside time [5]. Crown lengthening can be performed freehand or guided by a wax-up used to demarcate the gingivoplasty line, while the osteotomy levels depend totally on the practitioner's experience [6]. Numerous instruments have been proposed for simplifying crown lengthening and helping to achieve golden proportions; nonetheless, these instruments do not consider either the patient's facial features or the root length [6].

Recently, digital dentistry, with its equipment and software, has improved the ability to plan and predict outcomes in complex cases, thus reducing the overall treatment time and complications risks [7]. Intraoral and facial scanners are considered the workhorses of digital dental workflows [8]. This combination can digitize clinical reality and transfer it to dedicated design software, where any esthetic procedure can be studied meticulously during the planning phase [8, 9]. Numerous concepts that involve an interdisciplinary approach were created for an improved workflow in the esthetic zone, such as surgical implant guides [10] and facially driven crown lengthening guides [11].

From a clinical perspective, based on these tools the practitioner can communicate more effectively with both the patient and the dental technician [3, 11]. However, despite the technological advancement, till date, there are still some limitations when it comes to soft tissue position after a crown lengthening procedure [6]. As a matter of fact, to achieve a harmonious smile, the implant shoulder is typically placed 4 mm apically to the adjacent gingival crown's level [4]. This result is difficult to obtain when raising a flap during a crown lengthening; hence, the need for virtual visualization of the future position of the gingivo-dental complex.

The present paper describes a compromised anterior maxilla rehabilitation of a young patient suffering from tooth loss and altered passive eruption by means of a novel multipurpose surgical guide. To the authors' knowledge, this is the first reported case in the literature to utilize a fully digital workflow for the design and fabrication of a single, patient-specific surgical guide that integrates and directs multiple procedures simultaneously. Using advanced computer-aided design software, the guide was planned to define accurately the gingival and osseous resection levels for esthetic crown lengthening, guide anterior implant placement in harmony with the recontoured gingivo-osseous levels, and assist in the extraoral preshaping of a soft tissue substitute for volume augmentation. In addition, a computer-designed provisional restoration was fabricated prior to surgery, allowing for immediate temporization of the implant following placement. The case highlights the potential of digital technology to unify complex surgical procedures into a streamlined, precise, and esthetically optimized treatment protocol.

## 2. Case Presentation

A 24-year-old healthy male visited the clinic seeking esthetic improvement of his smile. His main complaints included a missing lateral incisor, excessive gingival display, brownish discoloration of the teeth, and altered chewing function due to the missing maxillary first molars. The patient had undergone radicular cyst enucleation and apicectomy 6 months prior in the maxillary anterior region.

**Figure 1:**
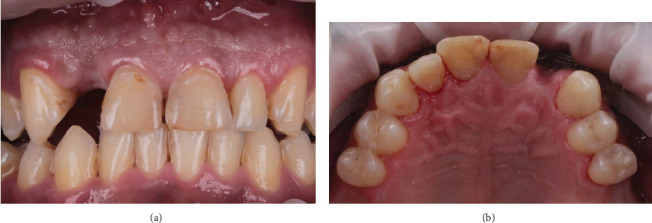
Intraoral photos showing the anterior maxilla: (a) Frontal view. (b) Occlusal view.

Clinically, the patient exhibited short, square-shaped anterior teeth and a high smile line that exposed a significant amount of gingiva (Figure 1a). The anterior teeth were previously restored with defective Class III composite fillings (Figure 1b). Periodontal probing tests were performed in order to confirm a potential altered passive eruption. A sulcus depth of 4 mm without bleeding or apparent attachment loss was observed; the cementoenamel junction (CEJ) was positioned more than 2 mm from the gingival margin, and the distance between the bone crest and CEJ was less than 1 mm, indicating an altered passive eruption of Type I-B by Coslet et al. [12].

The extracted lateral incisor zone showed a moderate horizontal tissue resorption (Figure 1b). Therefore, a cone beam computed tomography (CBCT) scan was performed to investigate the bone level around the anterior teeth and the possibility of placing implants. CBCT slices showed bone apposition around the teeth apices as a sign of postcyst enucleation healing, high bone levels around the teeth, and adequate bone dimensions for implant placement. Additionally, CBCT measurements of the bone-to-CEJ distance on the eight anterior teeth showed a distance less than 2 mm, confirming the clinical altered passive eruption diagnosis.

Based on these findings, the treatment plan of the patient consisted of a simultaneous guided implant placement and temporization in conjunction with a preprosthetic crown lengthening for the eight anterior teeth. Concerning the extracted lateral incisor site, it was decided to resolve the deficiency by the means of soft tissue augmentation. Following 3 months of healing, and depending on the extent of tooth structure loss, an appropriate type of prosthetic restoration will be selected for the six anterior teeth.

**Figure 2:**
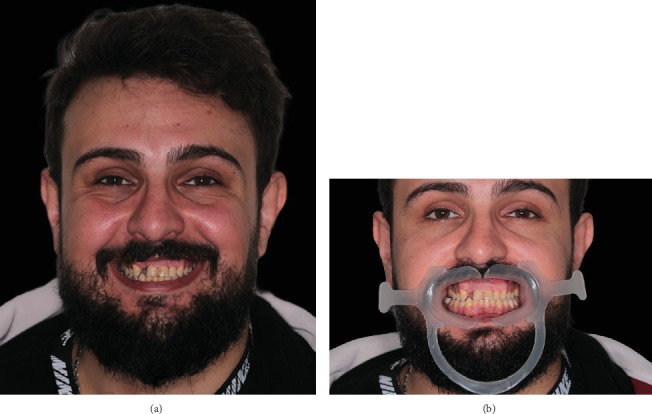
Extraoral photos of the patient. (a) Smiling. (b) With retractors in place.

**Figure 3:**
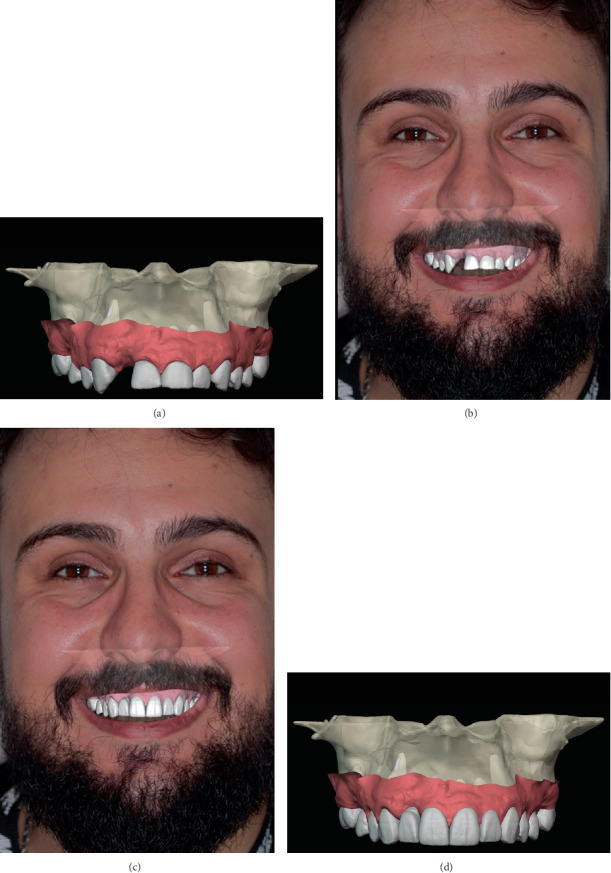
Virtual model preparation and smile design: (a) Super-imposed intraoral scan over the segmented bone model. (b) Virtual model over the patient extraoral photo. (c) Smile design wax-up over the patient extraoral photo. (d) Virtual model with the new smile design.

The necessary clinical data for the smile design were acquired through an intraoral scan (IOS) of the lower and upper jaw (R2I3; MegaGen, South Korea) and two-dimensional extraoral photos of the patient's face smiling and with the lips retracted (Figure 2a,b) (90D; Canon, Japan). A virtual model was made by importing the CBCT files to a planning software (Blue Sky Plan v4.12.13; Blue Sky Bio, United States) where an artificial intelligence-based segmentation for the hard tissue structures was performed; subsequently, the IOSs were imported and aligned with the segmentation model (Figure 3a). The segmentations and the scans were then modified on specialized software (Meshmixer; Autodesk research, United States) to obtain a complete anatomical model with the following segments: alveolar bone, root segmentations merged with the IOS data to have the root from the segmentation and the crown from the IOS. The gingival tissue was segmented and treated as a separate entity.

**Figure 4:**
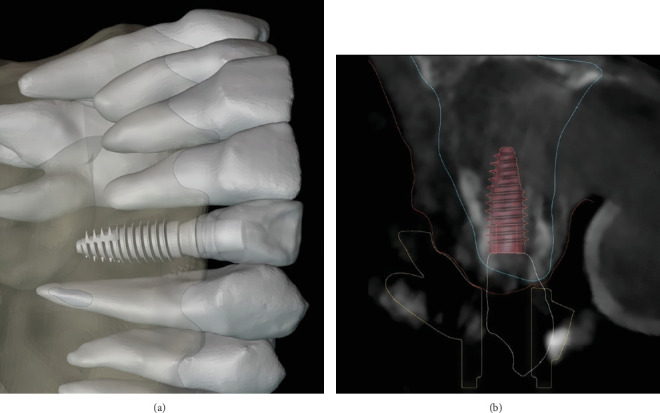
Prosthetically driven implant placement planning. (a) Digital simulation showing the position of the implant following crown lengthening bone margins. (b) Sagittal cone beam computed tomography slice showing the prosthetically driven implant position.

**Figure 5:**
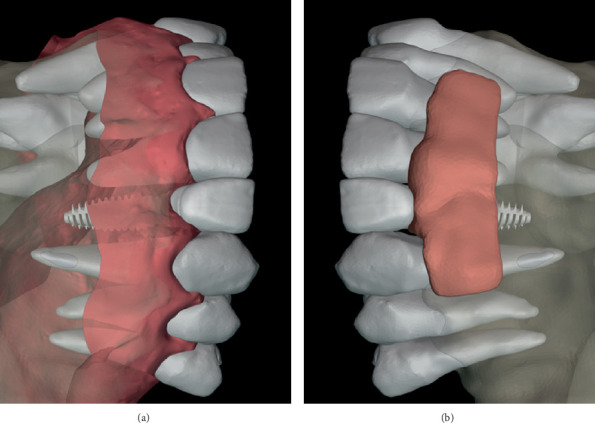
Soft tissue guide design. (a) Digital simulation picture showing the soft tissue deficiency at the mesiobuccal region of the lateral incisor. (b) The guide form and position according to the adjacent teeth.

**Figure 6:**
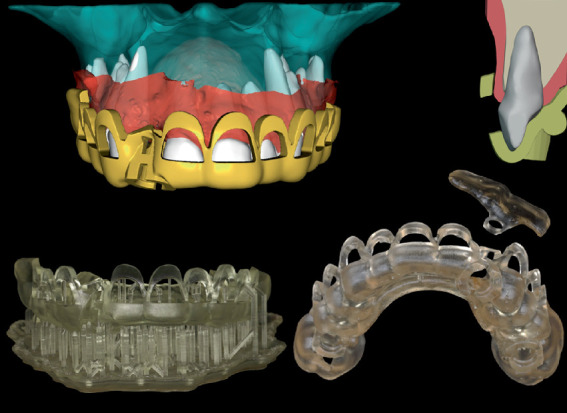
The “unique” surgical guide components.

The virtual models and extraoral photos were exported to another software for the digital smile design (DentalCAD 3.2; Exocad) (Figure 3b), and a wax-up featuring the new smile was created based on the patient's smile line, the position of the CEJ, and the remaining root structure after apicectomy (Figure 3c,d). The authors followed the digital workflow described earlier by Mendoza-Azpur et al. [13]. Following the smile design, the wax-up model was exported to an implant planning software (Blue Sky Plan v4.12.13; Blue Sky Bio, United States) where the three implants were placed in a prosthetically driven position. Care was given to place the anterior implant 4 mm below the created gingival level (Figure 4a,b) in an attempt to create an appropriate emergence profile. The provisional crown was shaped according to the previously set wax-up. Using the Meshmixer software, the potential area of the bone needing soft tissue grafting was selected and separated from the bone model. A 1-mm thickness was applied to this selection to create a solid three-dimensional printed object. Finally, a Boolean operation with the implant axis cylinder of 4 mm was performed to create the window in which the soft tissue graft will be secured within the implant (Figures 5 and 6). The simulation data were then exported to the guide designing software (blenderfordental; B4D, Australia). Concerning the guide design, buccal windows were created on the anterior teeth where the internal limit facilitates a precise gingivectomy, while the external part corresponds to the desired level of osteotomy (Figure 6). As for the implant placement, a sleeveless design allowing control of implant drilling and depth was created. The final STL file of the guide was then transferred to a slicing software and printed (form 3B printer; Formlabs, United States) using surgical guide resin. The postprocessing treatment of the guide consisted of immersion in an isopropyl alcohol bath for 20 min (Formwash; Formlabs, United States), followed by a 30-min period of 60° heated LED light curing bed (Formcure; Formlabs, United States). Finally, the surgical guide was inspected and autoclaved prior to the surgery.

**Figure 7:**
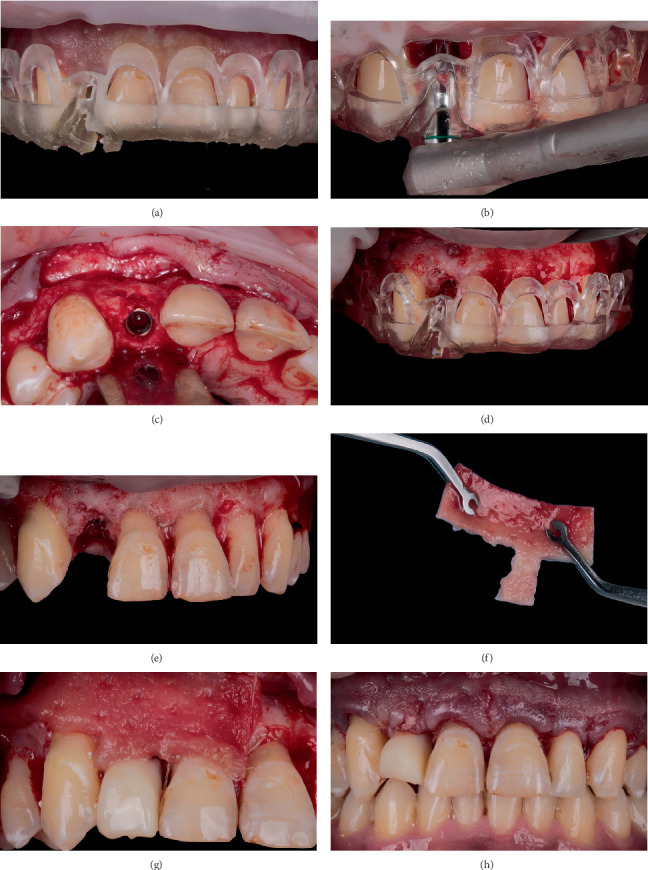
The surgical workflow. (a) The surgical guide in place. (b) Implant placement through the guide. (c) Implant position within the bone envelope. (d) Guided crown lengthening osteotomy. (e) Postosteotomy and implant placement. (f) The extraorally shaped soft tissue substitute. (g) Adaptation of the soft tissue graft to the recipient site. (h) Sutured flap.

Following local anesthesia infiltration, the guide was placed and gingival tissues enclosed within the guide's inner window were removed using a 15C blade (Figure 7a). Internal bevel intrasulcular incisions were performed including a midcrestal incision at the implant site, followed by two vertical releasing incisions placed at the distal aspects of the second premolars, resulting in a full-thickness trapezoidal flap design. The vertical components were intentionally positioned outside the visible smile zone to minimize the risk of postsurgical scarring in esthetically critical areas. A 3.7 × 10 mm deep threads bone level implant (Blue Diamond; MegaGen, South Korea) was placed in a fully guided fashion with an insertion torque of 70 N.cm (Figure 7b,c). Beveled osteotomy was performed following the guide's outer curvature and was carried out using the crown lengthening piezo inserts CE1 and CE3 (Satelec; Acteon, France) (Figure 7d,e). A collagen gingival substitute (Mucoderm; Botiss, Germany) infused with cross-linked hyaluronic acid (Hyadent BG, Regedent, Switzerland) for accelerated healing [14] was tailored extraorally according to the printed guide (Figure 7f) and fixed by means of surgical pins and periosteal sutures. The coronal portion of the matrix was perforated in a way to allow the potential abutment to emerge out of it (Figure 7g). Finally, the provisional was extraorally cemented onto a Ti-base and screwed in to guide and model the soft tissue healing. The flap was sutured using 5/0 resorbable sutures (Glycolon; Resorba, Germany) (Figure 7h). 4.1 × 8 mm implants was placed in a flapless fashion at the left and right first maxillary molar sites. The patient was given antibiotics for 7 days 2 g/day orally (Ospamox 1000 mg; Sandoz, Switzerland). Nonsteroidal anti-inflammatory drug (Brufen 400 mg; Abbott Laboratories, United States) three times daily for 3 days, along with a 0.12% chlorhexidine mouthwash three times daily for 2 weeks as postoperative medication.

**Figure 8:**
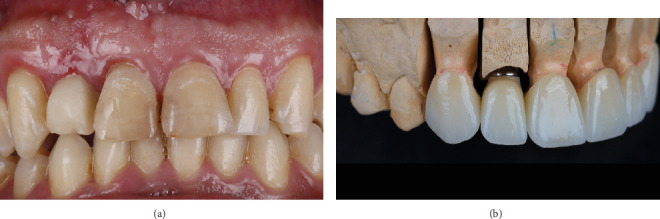
Prosthetic workflow. (a) The anterior zone at 3 months postoperatively. (b) Layered zirconia crowns on stone cast.

**Figure 9:**
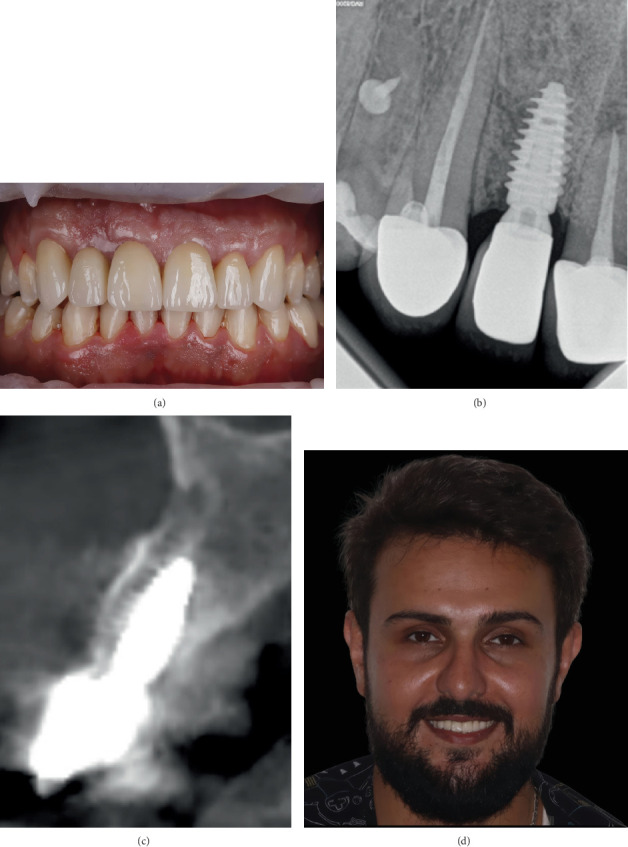
The postoperative situation. (a) Intraoral frontal photo showing the final esthetic outcome. (b) Peri-apical radiograph showing the area of the implant. (c) Sagittal cone beam computed tomography slices taken 12 months postoperatively. (d) Extraoral photo of the patient smiling.

Three months after the implant placement, the anterior region was ready to proceed with the prosthodontic phase (Figure 8a). Given the extent of dental tissue alterations, the choice of layered zirconia crowns seemed suitable. Following teeth preparation, an impression of the whole maxilla was submitted to the dental laboratory. The shade was chosen according to the adjacent teeth. A2 shaded zirconia cores (IPS e.max ZirCAD Prime; Ivoclar, Liechtenstein) were computer designed using DentalCAD 3.2 software. After the milling step, layers of aluminosilicate glass-ceramic (IPS Emax Ceram; Ivoclar, Liechtenstein) were added progressively to complete the fabrication of the crowns (Figure 8b). Finally, the layered crowns were cemented using a dual curing self-adhesive luting composite (Permacem; DMG, Germany) (Figure 9a). The concluding CBCT scan and peri-apical X-ray of the anterior region showed a healed surgical site and an ideal implant position within bone at the end of the treatment (Figure 9b,c). The extraoral photo of the patient indicated the resolution of the gummy smile when smiling (Figure 9d).

## 3. Discussion

Several challenges encountered in this case could not have been addressed efficiently without a multidisciplinary approach. The prior apicectomy expanded the available prosthodontic treatment options, while also reducing the amount of osteotomy required during the planned crown lengthening. From a periodontal perspective, the extent of osteotomy required during crown lengthening must be carefully calculated to maintain sufficient alveolar bone for optimal tooth function, while achieving the desired corrected gingival levels in the anterior region. At the implant site, proper platform positioning must correspond precisely to the gingival margins of the adjacent teeth following the crown lengthening procedure. When properly executed, these parameters provide a stable foundation for esthetic crown design.

In the present case, the digital workflow was the key to success, enabling virtual prediction of crown lengthening outcomes and allowing simultaneous implant placement. The integration of CBCT images, intraoral, and facial images within a single computer-aided design (CAD) platform enabled the evaluation of multiple treatment scenarios, facilitating the development of a patient-specific treatment plan. Another challenge was the need for multiple surgical interventions, which could potentially reduce patient motivation and compliance. Owing to the flexibility of CAD software, a unique multipurpose surgical guide was developed to consolidate the procedures into a single surgical intervention. The authors designed the “unique” surgical guide by combining features of previously established implant placement guides [10] and a dual crown lengthening guide [11] integrating the advantages of both for the sake of precise surgical tissue manipulation and reduced chair time. However, future studies should investigate the use of metal rather than resin for guide fabrication to create more durable osteotomy guides and minimize the dispersion of resin particles in the surgical site. Borham et al. [15] demonstrated that a CAD-CAM–based dual surgical guide significantly outperformed the conventional approach in terms of operating time and surgical efficiency (*p* < 0.001). Previously, Herrero et al. [16] figured out that nonguided conventional crown lengthening procedures often failed to achieve the intended 3 mm distance between the restoration margin and the alveolar crest, even if experienced periodontists were conducting the surgery. Upon literature review, only one technical note published in March 2024 by Rodeja-Vazquez et al. introducing a crown lengthening and posterior implant placement using a single guide was figured out, which, however, did include a clinical case [17]. Moreover, the latter technique required an additional clinical step involving the physical trial of a printed wax-up followed by rescanning for guide design. In the present paper, all the designing steps were performed digitally without the need for an additional chairside step.

## 4. Conclusions

Using latest technology data capturing and processing, the proposed treatment concept permits the practitioner to reduce multiple surgeries into one using a single surgical guide, hence saving both the practitioner and the patient from the burden of multiple surgeries.

## Data Availability

Data used to support the findings of this study are available from the corresponding author upon request.
